# Antimicrobial Stewardship Opportunities in Peripartum Patients: An Administrative Data Review

**DOI:** 10.1017/ash.2025.195

**Published:** 2025-09-24

**Authors:** Alexia Foy-Crowder, Jessica Britt, Sean Stuart, Joseph Kohn, Sarah Withers, Amy Crockett, Pam Bailey

**Affiliations:** 1 Prisma Health Midlands; 2 Prisma Health Upstate/University of South Carolina - Greenville; 3 University of South Carolina School of Medicine Greenville; 4 University of South Carolina School of Medicine Columbia

## Abstract

**Background:** There are ongoing significant increases in antimicrobial resistant infections in hospitalized patients in the United States, emphasizing the importance of antimicrobial stewardship initiatives like appropriate antimicrobial use and accurate laboratory detection of infections. The special population of obstetric patients has received relatively limited focus in prior reports about antimicrobial stewardship opportunities. **Methods:** A retrospective observational review was conducted through a single large healthcare system’s electronic medical record to evaluate antimicrobial use in peripartum patients, defined as 30 days pre- or post-delivery. Our hypothesis was that most antibiotic use could be attributed to American College of Gynecology (ACOG) recommended therapy for common situations such as group B Streptococcus (GBS) prophylaxis, surgical site infection (SSI) prophylaxis, or intra-amniotic infections (IAI). Data regarding antimicrobial allergies were also collected. **Results:** Between April 2018 and July 2024, 77,062 mother-baby dyads were identified. 40,576 (52.6%) had antimicrobial utilization peripartum. Redundant antimicrobial coverage was common; most commonly cefazolin and penicillin (n=1402) and cefazolin and ampicillin (n=675). A subanalysis of 8528 (11% total deliveries) patients receiving the most common antimicrobials demonstrated 199 separate regimens utilized, 92 (46.2%) of which had duplicative spectrum of activity. The top three regimens were cefazolin and penicillin (n=126), cefazolin and ampicillin (n=51), and cefazolin and cefoxitin (n=47). 33 (16.6%) were in line with ACOG guidelines for GBS or SSI prophylaxis or IAI. 12 of the 33 (36.4%) were ACOG endorsed regimens with duplicative spectrum of activity. Allergies were common in the subanalysis cohort; 3957 (46%) patients had penicillin allergies and 816 (9.5%) patients had cephalosporin allergies. **Conclusions:** An administrative review of peripartum antimicrobials indicates significant opportunities for antimicrobial stewardship, particularly around antimicrobial coverage for conditions for which there is overlapping spectrum of activity, such as GBS prophylaxis with SSI prophylaxis. There are also significant opportunities in delabeling penicillin and cephalosporin allergies as there is the lead time of the pregnancy, usually with multiple touchpoints with obstetric care providers, to explore the accuracy of the allergy label. Steps to improve antimicrobial utilization around guideline-concordant antimicrobials with overlapping spectrum of activity as well as delabeling antimicrobial allergies will lead to decreased variability in antimicrobial prescribing in this population.

